# Steady‐state imaging with inhomogeneous magnetization transfer contrast using multiband radiofrequency pulses

**DOI:** 10.1002/mrm.27984

**Published:** 2019-09-19

**Authors:** Shaihan J. Malik, Rui P. A. G. Teixeira, Daniel J. West, Tobias C. Wood, Joseph V. Hajnal

**Affiliations:** ^1^ School of Biomedical Engineering and Imaging Sciences King's College London London United Kingdom; ^2^ Centre for the Developing Brain King's College London London United Kingdom; ^3^ Neuroimaging Department Institute of Psychiatry, Psychology & Neuroscience King's College London London United Kingdom

**Keywords:** dipolar order, ihMT, inhomogeneous MT, magnetization transfer, myelin imaging

## Abstract

**Purpose:**

Inhomogeneous magnetization transfer (ihMT) is an emerging form of MRI contrast that may offer high specificity for myelinated tissue. Existing ihMT and pulsed MT sequences often use separate radiofrequency pulses for saturation and signal excitation. This study investigates the use of nonselective multiband radiofrequency pulses for simultaneous off‐resonance saturation and on‐resonance excitation specifically for generation of ihMT contrast within rapid steady‐state pulse sequences.

**Theory and Methods:**

A matrix‐based signal modeling approach was developed and applied for both balanced steady state free precession and spoiled gradient echo sequences, accounting specifically for multiband pulses. Phantom experiments were performed using a combination of balanced steady state free precession and spoiled gradient echo sequences, and compared with model fits. A human brain imaging exam was performed using balanced steady state free precession sequences to demonstrate the achieved contrast.

**Results:**

A simple signal model derived assuming instantaneous radiofrequency pulses was shown to agree well with full integration of the governing equations and provided fits to phantom data for materials with strong ihMT contrast (PL161 root mean square error = 0.9%, and hair conditioner root mean square error = 2.4%). In vivo ihMT ratio images showed the expected white matter contrast that has been seen by other ihMT investigations, and the observed ihMT ratios corresponded well with predictions.

**Conclusions:**

ihMT contrast can be generated by integrating multiband radiofrequency pulses directly into both spoiled gradient echo and balanced steady state free precession sequences, and the presented signal modeling approach can be used to understand the acquired signals.

## 1 INTRODUCTION

Inhomogeneous magnetization transfer (ihMT) is a method for generating contrast specific to substances with lamellar or otherwise ordered microstructure that can support local nonzero dipolar magnetization order.^1^, ^2^, ^3^ It has been demonstrated that this effect could have high specificity for myelinated tissue, and ihMT methods are now being established as structural markers for myelin^4^ with potential use for studying demyelinating conditions, such as multiple sclerosis.^5^

**Figure 1:**
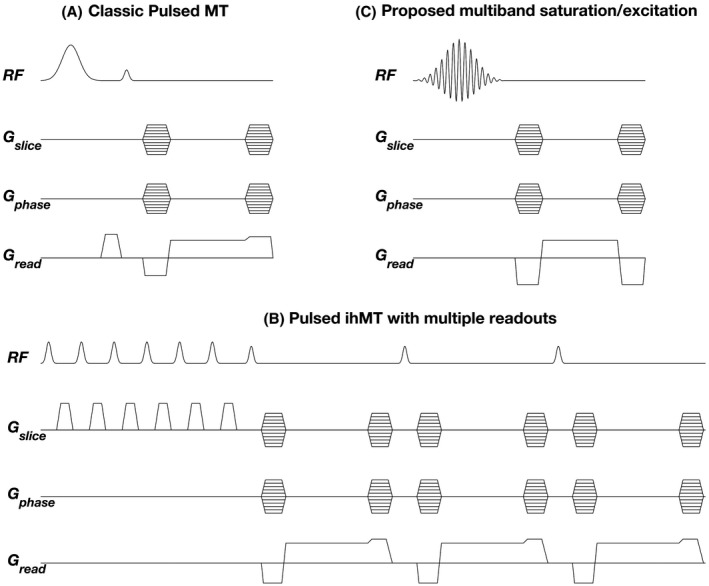
A, Classic pulsed MT weighted sequence containing off‐resonant saturation pulse followed by gradient echo readout. Here shown as a 3D sequence, although this can also be made slice selective. B, Pulsed ihMT sequence from Mchinda et al^7^ consisting of multiple saturation pulses using either fixed or alternating frequency offsets for generation of ihMT contrast, followed by multiple gradient echo readouts. C, Proposed sequence using nonselective multiband pulses that simultaneously saturate semisolid magnetization components while exciting water on‐resonance. This may be combined with either balanced (illustrated) or spoiled readouts. Each of these diagrams shows 1 TR period of a steady‐state sequence

The ihMT effect results in different observable MT characteristics when single or dual‐frequency off‐resonance irradiation are used during imaging. Novel pulse sequences incorporating either multiband or rapidly alternating single‐frequency saturation pulses have been proposed to generate ihMT weighted images^6^, ^7^ demonstrating strong white‐matter specificity. More recently, quantitative approaches to measure the dipolar relaxation time^8^ and influence of ihMT on free water T_1_ have also emerged. Existing work has focused on a preparation‐based paradigm in which saturation pulses are interleaved with readout periods for measurement, as illustrated by Figure 1. Classic pulsed MT sequences^9^ often consist of separate saturation and excitation pulses preceding a gradient echo readout (Figure 1A), while a recently proposed ihMT sequence^7^ uses multiple saturation pulses with fixed or alternating frequency offsets followed by multiple gradient echoes (Figure 1B). In our own previous work,^10^ we have developed nonselective multiband excitation pulses to control for MT effects in variable flip angle relaxometry, demonstrating that use of constant radiofrequency (RF) power over all flip angles leads to more stable relaxation time measurements. This sequence, illustrated in Figure 1C, is essentially a modified pulsed MT sequence with nonselective multiband pulses used to simultaneously perform saturation and excitation, and may be combined with a spoiled or balanced readout. Multiband pulses potentially lead to shorter repetition times (TRs) and include the flexibility to add either single or dual frequency saturation bands for generation of ihMT contrast.

In this work, we explore the use of the proposed multiband sequence for generation of ihMT contrast and formulate a matrix‐based signal model for ihMT that may be used to directly calculate steady‐state signals for spoiled gradient echo (SPGR) and balanced steady state free precession (bSSFP) sequences when dipolar terms are present and multiband pulses are used. We investigate the validity of this matrix‐based model, compare predictions with phantom experiments, and present in vivo ihMT ratio images formed using the proposed sequences.

## 2 THEORY

### 2.1 Bloch‐McConnell‐Provotorov equations

The commonly used “binary spin bath” model for magnetization transfer effects divides tissue magnetization between free water and semisolids, labeled with superscripts *f* and *s*, respectively. In this model, the (Zeeman) magnetization of the free water pool $\left[M_{x}^{f}\;M_{y}^{f}\;M_{z}^{f}\right]$ interacts with (Zeeman) magnetization of the semisolid pool, written $M_{Z}^{s}$. The semisolid pool has a very short transverse relaxation time ($T_{2}^{s}\approx 10\mu s$), so transverse components are typically excluded. The ihMT effect arises in some semisolids because of the presence of a dipolar interaction term (D) as well as Zeeman term (Z) in the quantum Hamiltonian. According to Provotorov theory as presented by Goldman,^11^ this may be described by subdividing the net magnetization in the semisolid pool between Zeeman and dipolar ordered pools $M_{Z}^{s}$ and $M_{D}^{s}$, respectively; here we use the formalism adopted by Lee et al^12^ to represent both terms as dimensionless polarizations. The magnetization of this system can be written as $M={\left[M_{x}^{f}\;M_{y}^{f}\;M_{z}^{f}\;M_{Z}^{s}\;M_{D}^{s}\right]}^{T}$. The model has been further updated by Varma et al^1^ to include multiple semisolid pools, some of which do not contain a dipolar order term. Varma et al^1^ showed that a model with two semisolid pools, one without and one with dipolar order (denoted s1 and s2, respectively) provides a good fit to experimental data. The overall state is, therefore, described by vector $M={\left[M_{x}^{f}\;M_{y}^{f}\;M_{z}^{f}\;M_{Z}^{s1}\;M_{Z}^{s2}\;M_{D}^{s2}\right]}^{T}$ whose temporal evolution is governed by:

(1) $$\frac{dM}{\mathrm{dt}}=\left(\Omega +\Lambda \right)M+\;C.$$

**Figure 2:**
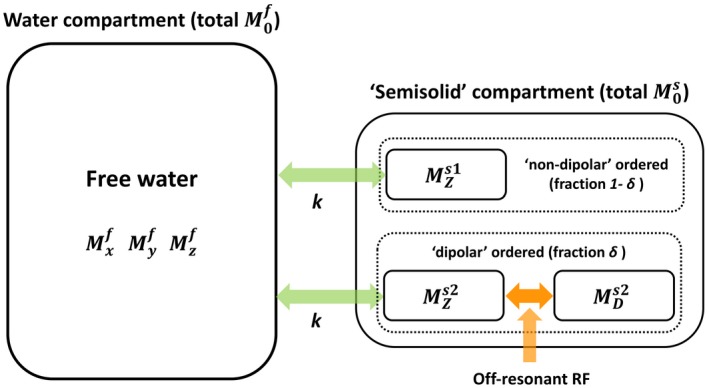
Multi‐compartment model consists of free water in contact with a semisolid compartment that has two sub‐parts s1 and s2. The former is a “classic” MT model with only Zeeman ordered magnetization; the latter contains a dipolar ordered component, which only exchanges with the Zeeman order under the action of off‐resonant RF saturation

Matrices Λ and C describe evolution in the absence of RF:

(2) $$\Lambda \;=\;\left(\begin{array}{cccccc} -R_{2}^{f} & \Delta \omega & 0 & 0 & 0 & 0\\ -\Delta \omega & -R_{2}^{f} & 0 & 0 & 0 & 0\\ 0 & 0 & -kM_{0}^{s}\;-R_{1}^{f} & kM_{0}^{f} & kM_{0}^{f} & 0\\ 0 & 0 & k\left(1-\delta \right)M_{0}^{s} & -kM_{0}^{f}\;-R_{1Z}^{s} & 0 & 0\\ 0 & 0 & k\delta M_{0}^{s} & 0 & -kM_{0}^{f}\;-R_{1Z}^{s} & 0\\ 0 & 0 & 0 & 0 & 0 & -R_{1D}^{s} \end{array}\right)C\;=\;\left(\begin{array}{c} 0\\ 0\\ R_{1}^{f}M_{0}^{f}\\ R_{1Z}^{s}\;\left(1-\delta \right)\;M_{0}^{s}\\ R_{1Z}^{s}\delta M_{0}^{s}\\ 0 \end{array}\right)$$

where $R_{1,2}^{f}$ are the free water relaxation rates (inverse of relaxation times); $R_{1Z}^{s}$ and $R_{1D}^{s}$ are semisolid Zeeman and dipolar relaxation rates, respectively; Δω is the off‐resonance frequency; *k* is the exchange rate constant between free water and semisolid pools; and $M_{0}^{f}$ and $M_{0}^{s}$ are thermal equilibrium magnetizations for the free and semisolid pools, respectively. Note that, in this article, *k* represents the intrinsic exchange rate as opposed to a directional rate (i.e., forward or reverse) as sometimes used in the literature (for example, see Yarnykh).^13^ For simplicity, we assume that *k* and $R_{1Z}^{s}$ are the same for pools s1 and s2, and use this formulation in this article. A more general version is given in the Appendix. Pools s1 and s2 occupy fractions 1-δ and δ, respectively, of the total semisolid compartment $M_{0}^{s}$ and we adopt the convention $M_{0}^{f}+M_{0}^{s}=1$. Figure 2 illustrates the relationships between compartments in this model.

To handle multiband pulses, we explicitly decompose them into contributions from separate frequency bands:

(3) $$B_{1}\left(t\right)=\sum_{j=1}^{N_{\mathrm{bands}}}b\left\{\Delta_{j};t\right\}e^{i\Delta_{j}t}$$

where $\Delta_{j}$ is the (angular) frequency offset of the *j* ^th^ band and $b\{\Delta_{j};\;t\}$ is the time domain pulse waveform associated with component frequency $\Delta_{j}$. It then follows that matrix Ω describing interactions due to RF pulses may be written as:

(4) $$\begin{array}{cc} \Omega =\left(\begin{array}{cc} \Omega_{\mathrm{free}} & \begin{array}{ccc} 0 & 0 & 0\\ 0 & 0 & 0\\ 0 & 0 & 0 \end{array}\\ \begin{array}{ccc} 0 & 0 & 0\\ 0 & 0 & 0\\ 0 & 0 & 0 \end{array} & \Omega_{\mathrm{semi}} \end{array}\right) & \Omega_{\mathrm{free}}=\left(\begin{array}{ccc} 0 & 0 & -\gamma B_{1,y}\\ 0 & 0 & \gamma B_{1,x}\\ \gamma B_{1,y} & -\gamma B_{1,x} & 0 \end{array}\right)\Omega_{\mathrm{semi}}=\sum_{j\;=1}^{N_{\mathrm{bands}}}\left(\begin{array}{ccc} -W_{j} & 0 & 0\\ 0 & -W_{j} & W_{j}\frac{\Delta_{j}^{\prime }}{\omega_{\mathrm{loc}}}\\ 0 & W_{j}\frac{\Delta_{j}^{\prime }}{\omega_{\mathrm{loc}}} & -W_{j}\;{\left(\frac{\Delta_{j}^{\prime }}{\omega_{\mathrm{loc}}}\right)}^{2} \end{array}\right) \end{array}$$

$\Omega_{\mathrm{free}}$ is simply the Bloch equation for the free pool. For the multiband pulses used in this work, only the on‐resonance component b{0;t} need be considered. $\Omega_{\mathrm{semi}}$ describes absorption by the semisolid pools and is explicitly written as a sum over frequency bands as suggested by Goldman^11^ for the case of “fast modulation”. The RF saturation rate is defined as $W_{j}=\pi \gamma^{2}{\left|b\{\Delta_{j}\}\right|}^{2}g\left(\Delta_{j}^{\prime },\;T_{2}^{s}\right)$, where $g\left(\Delta ,\;T_{2}^{s}\right)$ is the absorption lineshape of the semisolid and $T_{2}^{s}$ is its transverse relaxation time. The term $\Delta_{j}^{\prime }\equiv \Delta_{j}-\Delta \omega$ accounts for off‐resonance effects on the local absorption response; for simplicity, these have been neglected in this work and we have instead assumed that $\Delta_{j}^{\prime }\approx \Delta_{j}$. The term $\omega_{\mathrm{loc}}$ characterizes the strength of local field fluctuations, which relate to dipolar interactions; this may be derived from the second moment of the lineshape.^1^, ^14^ As with exchange and relaxation rates, we assume both semisolid compartments have the same absorption lineshape.

There are a few properties to note for sequence modeling: (i) the first element of $\Omega_{\mathrm{semi}}$ is exactly as commonly used to describe “classic” MT effects; (ii) exchange between $M_{Z}^{s2}$ and $M_{D}^{s2}$ is purely RF driven, while exchange between $M_{z}^{f}$ and $M_{Z}^{s1,2}$ occurs continuously; (iii) coupling terms in $\Omega_{\mathrm{semi}}$ are linear in $\Delta_{j}$ so equal off‐resonance RF power applied simultaneously at ±Δ (or at Δ=0) eliminates mixing between Zeeman and Dipolar components of s2 (this is the ihMT effect); (iv) $M_{D}^{s2}$ relaxes to a thermal equilibrium value of zero.

### 2.2 MRI signal simulations

The system of differential equations described above, referred to here as the Bloch‐McConnell‐Provotorov (BMP) equations, allow for MRI signal modeling of this system with arbitrary pulse sequences. Here, we consider rapid gradient echo sequences and make the common approximation that RF pulses act instantaneously. Under this approximation, the evolution during each pulse is $\dot{M}=\left\langle \Omega \right\rangle M$ where ⟨Ω⟩ is the time average of Ω over pulse duration τ, with solution $M\left(t+\tau \right)=RM\left(t\right)$ where $R=\exp\left(\langle \Omega \rangle \tau \right)$. The matrix exponential preserves the block diagonal form of Ω such that R consists of the normal rotation matrix for the free water in the upper left block, and a saturation matrix for the semisolid pool according to the mean square B_1_ of the pulse in the lower right block; this is exactly the approach taken for modeling of “classic” MT in rapid sequences.^15^ Evolution in the absence of RF is governed by $\dot{M}=\Lambda M+C$, which has the general solution $M\left(t+TR\right)=SM\left(t\right)+\left(S-1\right)\Lambda^{-1}C$ for time period TR, where $S=\exp\left(\Lambda \mathrm{TR}\right)$. It is hence possible to write the following signal expressions for SPGR:

(5) $$M^{SPGR}={\left(I-R\Phi S\right)}^{-1}R\Phi \left(S-I\right)\Lambda^{-1}C$$

and bSSFP:

(6) $$M^{bSSFP}={S^{1/2}\left(\Phi -RS\right)}^{-1}R\left(S-I\right)\Lambda^{-1}C.$$

The matrix Φ accounts for spoiling or phase cycling in each case. For SPGR, we assume perfect spoiling of transverse magnetization and so Φ=diag[001111] is used to remove transverse components after each TR; for bSSFP, we assume π phase cycling between successive pulses, hence Φ=diag[-1-11111]. The expression for SPGR gives the magnetization immediately after an RF pulse, while for bSSFP it is at the midpoint between 2 RF pulses. Equations 5 and 6 are generalizations of standard steady‐state equations used for these sequences and give the same predictions as $M_{0}^{s}\to 0$. Given that dipolar relaxation rates ($R_{1D}^{s}$) are often very fast (time constants <10ms), we have also examined the validity of treating RF pulses instantaneously by comparing with full time integration of the BMP equations.

### 2.3 Time integration of BMP equations

Time integration for these sequences is cumbersome because hundreds of TR periods are required to reach a steady state, so a more direct method was devised. Equation 1 is first written as $\dot{M}=AM+C$, and then reformulated as:

$$\dot{\tilde{M}}=\tilde{A}\tilde{M}$$

(7) $$\begin{array}{cc} \tilde{M}\;=\;\left[\begin{array}{c} M\\ 1 \end{array}\right] & \tilde{A}\;=\;\left[\begin{array}{ccc} A & & C\\ & 0 \end{array}\right] \end{array}$$

where “1” is a scalar value, and “0” represents a row vector of zeroes matching the dimensionality of **A** and **C** ([0 0 0 0 0 0 0] in this case). The advantage of writing in this way is that the differential equation is homogeneous and the solution at any small time increment during which $\tilde{A}$ is constant is $\tilde{M}\left(t+\Delta t\right)=\exp\left(\tilde{A}\left(t\right)\Delta t\right)\tilde{M}\left(t\right)$. This may now be integrated over one TR period, with a small time‐step Δt allowing the shape of the RF pulse to be accounted for. The result is a single matrix that describes the evolution, such that:

$$\tilde{M}\left(t+TR\right)=\tilde{X}\tilde{M}\left(t\right)$$

(8) $$\tilde{X}=\prod_{n=1}^{N}\exp\left(\tilde{A}\left(n\Delta t\right)\Delta t\right)$$

where n is an index and N=TR/Δt. The product must be evaluated by left multiplication of successive matrices. Phase cycling or forced “perfect” transverse spoiling can be added by incorporating matrix Φ into the product. The steady state solution requires that $\tilde{M}$ is invariant after multiplication by $\tilde{X}$— i.e., it is the eigenvector of $\tilde{X}$ with an associated eigenvalue of 1. To remain consistent with Equations 5 and 6, the integration is performed starting at the end of an RF pulse for SPGR, and starting at the middle of a TR period for bSSFP. As outlined above, $\Omega_{\mathrm{semi}}$ is evaluated by considering the time domain representation of the individual frequency bands $b\{\Delta_{j};t\}$ that compose the multiband pulses.

## 3 METHODS

**Figure 3:**
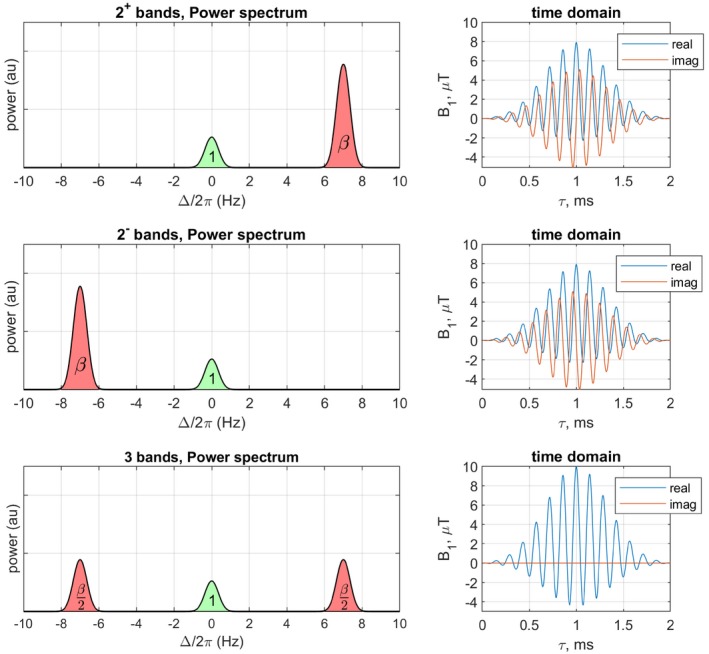
Illustration of the multiband excitation pulses used in this work. All are spatially nonselective and have the same on‐resonance contribution (shaded green on power spectra), which is responsible for flipping the free water magnetization. The off‐resonant components illustrated have the same total power (red shaded area) and this can be split either between 2 symmetric bands, or 1 band with either a positive or negative frequency offset. Time domain profiles show that the 3‐band pulses have the higher peak B_1_, as expected from Equation 10C

In this article we explore the use of nonselective multiband RF pulses (as proposed by Teixeira et al^10^) with either 2 or 3 bands, to generate ihMT contrast in 3D gradient echo sequences. In all cases, the pulses have an on‐resonance band responsible for flipping the free water magnetization; the 2‐band pulses have a single off‐resonance saturation band, whereas 3‐band pulses have symmetric off‐resonant bands (see Figure 3). Using these pulses, steady state solutions (Equations 5 and 6) are compared with time integration of the BMP equations (see Section 2.3), and expected contrast levels are explored for a range of tissue parameters taken from literature. Predictions are compared with phantom measurements, and in vivo images are also presented. Matlab code required for RF pulse generation and signal simulations has been posted at https://github.com/mriphysics/ihMT_steadystate, (hash 0db78e5 was used for the presented results). Phantom measurements are also available at the same site.

### 3.1 Multiband RF pulse design

Multiband pulses with power spectra illustrated by Figure 3 can be generated by multiplying single‐band on‐resonance pulse b{0;t} by modulation function $\omega_{\mathrm{mod}}\left(t\right)$. A key parameter for quantifying saturation power is the mean square B_1_ (${\langle B}_{1}^{2}\rangle$)^6^, ^7^ because this (or the root mean square, $B_{1}^{\mathrm{rms}}$) is a parameter easily accessible on many clinical MR systems. An SPGR or bSSFP sequence using single band excitation pulse b{0;t} has mean‐square B_1_ given by

(9) $${\langle B}_{1}^{2}\left(0\right)\rangle =\frac{1}{\mathrm{TR}}\int_{0}^{\tau }b{\left\{0;\;t\right\}}^{2}dt$$

where τ is the pulse duration. This can be turned into the required multiband pulse using modulation functions defined as follows:

(10a) $$\begin{array}{c} 2\;\text{bands with offset}\;+\Delta :\\ \;\;\;\omega_{\mathrm{mod}}\left(t\right)=1+\sqrt{\beta }\left(\cos\Delta t+i\sin\Delta t\right) \end{array}$$

(10b) $$\begin{array}{c} 2\;\text{bands with offset}\;-\Delta :\\ \;\;\;\omega_{\mathrm{mod}}\left(t\right)=1+\sqrt{\beta }\left(\cos\Delta t-i\sin\Delta t\right) \end{array}$$

(10c) $$\begin{array}{c} 3\;\text{bands with offsets}\;\pm \Delta :\\ \;\;\;\omega_{\mathrm{mod}}\left(t\right)=1+\sqrt{2\beta }\cos\Delta t \end{array}$$

where β quantifies the ratio of off‐resonant to on‐resonant power, calculated by

(10) $$\beta \equiv {{(\langle B}_{1}^{2}\rangle }^{\mathrm{TOT}}-{\langle B}_{1}^{2}\left(0\right)\rangle )/{\langle B}_{1}^{2}\left(0\right)\rangle$$

and ${{\langle B}_{1}^{2}\rangle }^{\mathrm{TOT}}$ is the desired total mean square B_1_ such that the whole sequence $B_{1}^{\mathrm{rms}}\equiv \surd {{\langle B}_{1}^{2}\rangle }^{\mathrm{TOT}}$. These expressions allow flexible design of multiband pulses that reach a required target total $B_{1}^{\mathrm{rms}}$ within a given pulse sequence; this depends on the properties of the pulse but also the sequence TR. All presented data used Gaussian pulse shapes for the single band pulses with time‐bandwidth‐product 2.26. For brevity, we will refer to single band pulses as 1B, 2 bands as 2B (or 2^+^B/2^−^B if distinction between sign of offset is important) and 3 bands as 3B. Function *gen_MB_pulse.m* in the accompanying code is an example implementation for generating these.

### 3.2 Numerical simulations

Signal simulations were performed for white matter using parameters based on measurements of human internal capsule taken from Mchinda et al^7^ relevant for 1.5T imaging: $R_{1}^{f}=1.54\;s^{-1}$, $R_{1Z}^{s}=1s^{-1}$, $R_{1D}^{s}=154\;s^{-1}$, $T_{2}^{s}=12.5\mu s$, $k=65s^{-1}$, $M_{0}^{s}=0.147$, δ=0.65; $R_{2}^{f}=12.5\;s^{-1}$ was taken from Sled and Pike.^9^ A Super‐Lorentzian absorption line‐shape^14^ was assumed for white matter. Because on‐resonance absorption is included in the model, the on‐resonance singularity in the Super‐Lorentzian function was handled by extrapolating between ±1 kHz as proposed by Bieri and Scheffler.^16^

#### 3.2.1 ihMT contrast from multiband steady‐state imaging

Signal simulations were performed for short TR sequences using 1B, 2B, and 3B pulses and a range of other sequence parameters (τ, TR, $B_{1}^{\mathrm{rms}}$, flip angle, Δ). The effect of long‐term dipolar order was assessed by modifying the white matter parameters to include a case with δ=0 (no “dipolar” pool) and δ=1 (whole semisolid pool contains “dipolar” component). The “ihMT difference” is defined as $\Delta ihMT=S_{2B}-S_{3B}$ where $S_{2B}$ is the signal from the 2B acquisition, etc. The “ihMT ratio” is then defined as $ihMTR=\frac{\Delta ihMT}{S_{1B}}$; similarly the MT ratio is defined as $MTR=\frac{S_{1B}-S_{2B}}{S_{1B}}$. To account for asymmetry induced by chemical shift of the semisolid line, the definition $S_{2B}=\frac{1}{2}(S_{2^{+}B}+S_{2^{-}B})$ is used for analysis of in vivo data. This is not necessary for numerical simulations because the line is centered on the water resonance in simulations. Because the single band images $S_{1B}$ are used as a reference for calculating both MTR and ihMTR, it is important to note that these images will also contain on‐resonance MT effects (e.g., see Gloor et al,^17^) although the effect is small for the sequences used in this work. This is inherently included in our calculations because the sum in Equation 4 includes the on‐resonance band (i.e., Δ=0). In general, the contrast in the $S_{1B}$ image is influenced by MT and obviously also by free water relaxation effects. It should not necessarily be expected that MTR and ihMTR measured by steady‐state sequences would be directly comparable to those obtained by other methods.

#### 3.2.2 Instantaneous RF approximation

The sequences used in this work typically used RF pulse durations that are not short with respect to the TR. Hence, the steady‐state signal expressions Equations 5 and 6 were compared with time integration using the method described in Section 2.3. Numerical integration used step size Δt=10μs. Simulations were performed for the white matter tissue parameters, except that $T_{1D}^{s}\equiv 1/R_{1D}^{s}$ was varied logarithmically from 0.1 ms to 10 ms. Balanced SSFP and SPGR sequences with TR = 5 ms and $B_{1}^{\mathrm{rms}}=5\mu T$ were simulated for 1B, 2B, and 3B pulses for durations varying between 0.4 ms and 2.5 ms.

Bieri and Scheffler^18^ proposed a correction to the “instantaneous approximation” bSSFP signal model for finite duration RF pulses, which amounts to a correction to R_2_ to account for the fact that magnetization does not spend the full TR period in the transverse plane. This correction was also implemented and compared with full time integration. The original correction method is for a single pool model and amounts to modifying R_2_ as a function of the true R_1_ and R_2_ and sequence properties. For our multi‐compartment model, we use the same formulation but substitute $R_{1,2}$ for $R_{1,2}^{f}$ in the expressions derived by Bieri and Scheffler.^18^

### 3.3 Phantom experiments

A phantom consisting of 4 sample tubes immersed in a bath of water for susceptibility matching was imaged on a Philips (Best, Netherlands) 1.5T Ingenia MR system. The samples included: water doped with MnCl_2_ (~0.01 mM); chemically cross‐linked bovine serum albumin (BSA, 10% w/w in water prepared as in Koenig et al^19^); prolipid 161 (PL161, 15% w/w; Ashland Inc, Covington, KY; prepared as in Swanson et al^2^ but excluding any T_1_ reducing agent); and off the shelf hair conditioner (HC, TRESemmé, Unilever PLC, London, UK). The MnCl_2_ phantom is not expected to exhibit any MT effect; BSA is a model substance for human tissue MT effects,^19^ but is not expected to exhibit ihMT contrast because of protein cross‐linking. PL161^2^, ^3^ and HC^8^, ^20^ have both been shown to exhibit strong MT and ihMT contrast as they contain lamellar liquid crystal structures.

The phantom was imaged using a series of bSSFP and SPGR sequences, all with TR = 5 ms, echo time = 2.5 ms, τ = 2.2 ms, Δ/2π = 8 kHz, isotropic resolution 1.5 × 1.5 × 1.5 mm^3^, no parallel imaging acceleration, time per scan 57 s. A total of 64 images were obtained using flip angles 10° to 80° (steps of 10°) for bSSFP, and 2° to 16° (steps of 2°) for SPGR, and then for each of these using 1B, 2^+^B, 2^−^B, and 3B excitation pulses. All multiband pulses were computed for $B_{1}^{\mathrm{rms}}=4.2\mu T$, whereas the single band pulses used only the on‐resonance component of these pulses, and so had variable $B_{1}^{\mathrm{rms}}$ over the range of flip angles. The data were analyzed by averaging the signal from each sample tube over a range of 45 mm along the length of each tube; segmentation was performed automatically using k‐means clustering using all images as the feature space.

The signal models (Equations 5 and 6) were then fitted to these averaged measurements to determine best‐fit model parameters for each sample. For each sample, data from all 64 images were fitted together as a single model fit with a common scaling factor added to account for unknown overall image scaling. Signals $S_{2^{+}B}$ and $S_{2^{-}B}$ were averaged before fitting because the model does not distinguish these. For SPGR sequences, Equation 5 was multiplied by an additional factor $e^{-TER_{2}^{f}}$ to account for transverse relaxation at the echo time, here using the approximation that $R_{2}^{f*}\approx R_{2}^{f}$. For bSSFP sequences, the Bieri‐Scheffler correction for finite pulse duration was applied. Matlab's *fmincon* optimization function was used with the interior point algorithm selected. Upper and lower bounds were used to constrain fits to plausible values; in each case, a range of bounds and initial starting points were tried out and the best fitting solutions in terms of normalized root mean square error (NRMSE) are reported. Uncertainties were computed by residual bootstrapping using 500 re‐samplings for each fit. All phantom data were fit using a Gaussian lineshape function, because this was empirically found to yield the lowest NRMSE in all cases. A full set of fitting bounds used for generating the presented data is given in Supporting Information Table S1, which is available online.

### 3.4 In vivo imaging

A single adult normal volunteer (age 24 years, male) was imaged on the same Philips 1.5T MR system, after giving written informed consent in line with local governance rules. Balanced SSFP images were acquired with flip angle 30°, TR = 5 ms, echo time = 2.5 ms, τ = 2.3 ms,Δ/2π = 8 kHz, isotropic resolution 1.5 × 1.5 × 1.5 mm^3^, matrix 151 × 143 × 93, 9 signal averages, no parallel imaging acceleration, time per scan 9 min. A standard Philips 32 element head receiver was used. Separate images were acquired with 1B, 2^+^B, 2^−^B, and 3B excitation pulses. $B_{1}^{\mathrm{rms}}$ was 4.73μT for all multiband sequences and 0.76μT for single band; hence, the off‐resonance $B_{1}^{\mathrm{rms}}$ for multiband sequences was 4.67μT because the RMS values add in quadrature. Images were registered and brain extracted using FSL FLIRT and BET tools, respectively,^21^, ^22^ before MTR and ihMTR were calculated. MTR and ihMTR images were smoothed with 3D Gaussian kernel, standard deviation 1 mm.

## 4 RESULTS

### 4.1 Expected signal curves and instantaneous pulse approximation

**Figure 4:**
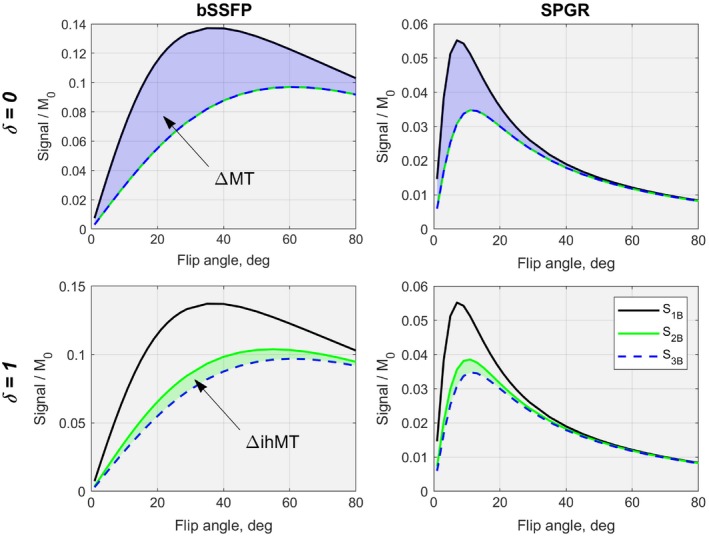
Simulated signal curves for bSSFP and SPGR sequences for the model white matter tissue parameters, with δ set to either 0 or 1. When δ = 0 there is no dipolar ordered fraction and so signals from 2B and 3B pulses (S_2B_ & S_3B_) are expected to be the same. However, both are significantly attenuated in comparison with single band signal S_1B_; this is the classic MT effect, shaded blue on the plots. When δ = 1 the whole semisolid compartment is coupled to a dipolar fraction and there is a difference between S_2B_ and S_3B_ in this case of around 10% of S_1B_; this is the inhomogeneous MT effect, shaded green

Figure 4 shows the predicted signals for the proposed sequences with TR = 5 ms, τ = 2 ms, Δ/2π = 7 kHz, $B_{1}^{\mathrm{rms}}=5\mu T$ over a range of flip angles (as described above the single band pulses have variable $B_{1}^{\mathrm{rms}}$). The “white matter” tissue parameters were used except that δ was set to 0 and 1 to examine extreme cases. For δ = 0, we see no difference between 2 or 3 band RF excitation; both lead to a strong reduction of signal compared with single band excitation, shaded blue. When δ = 1, signal $S_{3B}$ is clearly lower than $S_{2B}$. This is expected because the 3B excitation decouples the dipolar ordered pool, leading to more efficient saturation of the semisolid Zeeman magnetization. Supporting Information Figure S1 plots ΔihMT and ihMTR as functions of flip angle for the δ=1 case, showing that ihMTR is predicted to be in the order of 10% for bSSFP at lower flip angles, and similar for the SPGR around the Ernst angle.

**Figure 5:**
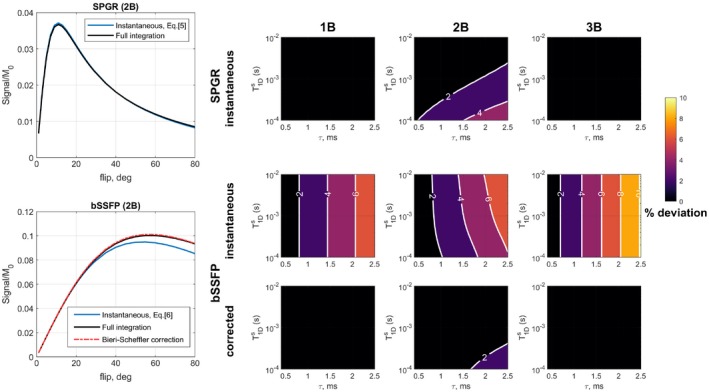
Examination of the instantaneous pulse approximation compared with full time integration. Left, Example SPGR and bSSFP curves for $B_{1}^{\mathrm{rms}}=5\mu T$, TR = 5 ms, τ = 2 ms, Δ/2π = 7 kHz for 2B pulses. The bSSFP curve without Bieri‐Scheffler correction diverges from the full integration curve at higher flip angles. Right, Percentage deviation between instant approximation and full integration for 1B, 2B, and 3B pulses with different τ and $T_{1D}^{s}$, flip angle = 60° for bSSFP and 7.1° (Ernst angle) for SPGR. Larger deviations are observed for 2 band pulses with short $T_{1D}^{s}$ and longer τ, and bSSFP without Bieri‐Scheffler correction

Figure 5 summarizes the comparison between the instantaneous pulse approximation and full integration methods described in Section 3.2.2. The left panels show example signal curves for 2 band excitation with $B_{1}^{\mathrm{rms}}=5\mu T$, TR = 5 ms, τ = 2 ms, Δ/2π = 7 kHz, using white matter tissue parameters. Results are very similar for SPGR, but systematic differences exist for bSSFP that are resolved with application of the Bieri‐Scheffler correction. The right‐hand panels of Figure 5 explore the effect of making the instantaneous assumption by displaying the percentage deviation between instantaneous and full integration methods, relative to the full integration result. For 1B and 3B, large deviations only exist for bSSFP without the Bieri‐Scheffler correction. For 2B excitation differences are present for both SPGR (up to 4.8%) and corrected‐bSSFP (up to 2.4%) in the case of short $T_{1D}^{s}$ (<1 ms) and long τ. The Bieri‐Scheffler correction was hence applied to all other bSSFP signal predictions presented in this study including Figure 4.

### 4.2 Phantom experiments

**Figure 6:**
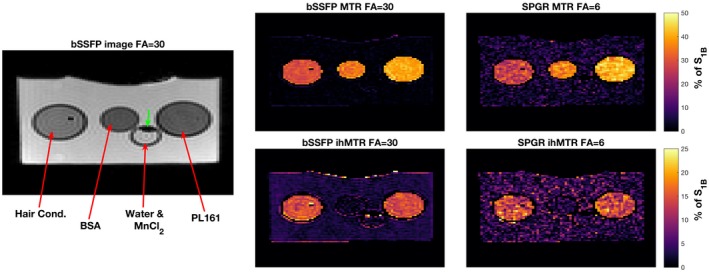
Phantom experiment results. Left, bSSFP image for 30° flip angle (1B) showing the phantom. Green arrow is marking the position of an air bubble in the MnCl_2_ phantom. Right, images of MTR and ihMTR as defined in the text, for 30° flip angle for bSSFP and 6° flip angle for SPGR. All MTR and ihMTRs are quoted in percentage units using the relevant single band image as a reference. Notably, the BSA phantom has a strong MTR but no ihMTR, while the PL161 and HC show both effects

**Figure 7:**
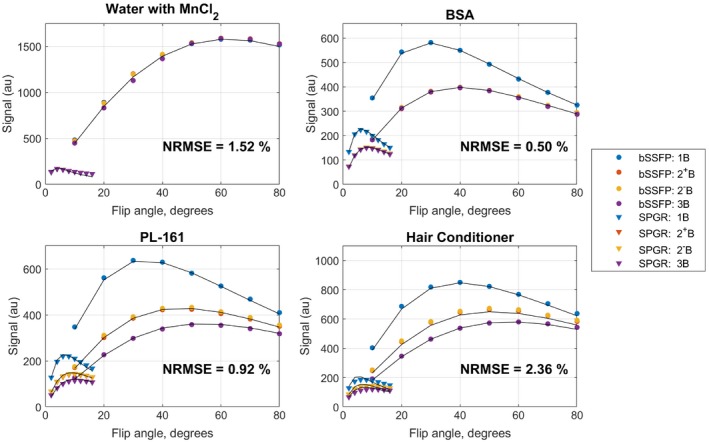
Fits to data (details in text, fitted parameter values in Table 1). In each case, all acquired datapoints were fitted to the model with a single parameter set. Excellent fits were obtained for water, BSA, and PL‐161 but for HC some error was seen in the SPGR fit. BSA shows no ihMT effect (2B & 3B identical), and in all cases 2^+^B and 2^−^B are very similar, suggesting little to no MT asymmetry in any sample. Signal values are as reconstructed by the scanner (arbitrary units) but are consistent between plots

Figure 6 shows the phantom imaging results with MTR and ihMTR calculated for single example flip angles from each image series (30° for bSSFP, 6° for SPGR). The BSA, HC, and PL161 samples all show an MTR of up to 50% for both sequences, while the water bath and MnCl_2_ tube do not. The HC and PL161 samples additionally show an ihMTR of around 15%, suggesting they exhibit dipolar order effects. Figure 7 shows the model fits to data with the fitted parameters given in Table 1. The MnCl_2_ phantom is expected to show no difference between any of the excitation pulse types and the best fit was obtained with $M_{0}^{s}$ fixed at zero. Small discrepancies in the bSSFP data can be attributed to off‐resonance distortion from an air bubble in this sample, visible on Figure 6.

**Table 1:** Fitted parameters for phantom experiments^a^

	$T_{1}^{f}$ (ms)	$T_{2}^{f}$ (ms)	$M_{0}^{s}$	$T_{1Z}^{s}$ (ms)	$T_{1D}^{s}$ (ms)	$T_{2}^{s}$ (µs)	*k* (s^‐1^)	δ
MnCl_2_	2174 ± 152	683 ± 45	0 (fixed)	–	–	–	–	–
BSA	1643 ± 29	53.2 ± 0.1	0.075 ± 0.001	130 ± 0	–	17.6 ± 0.0	70.1 ± 0.0	0 (fixed)
PL161	1912 ± 80	72.8 ± 0.4	0.150 ± 0.003	202 ± 2	20.7 ± 0.0	17.5 ± 0.1	46.7 ± 0.0	1 (fixed)
HC	1975 ± 127	158 ± 4	0.060 ± 0.004	189 ± 6	23.3 ± 0.1	19.2 ± 0.1	67.8 ± 0.1	0.683 ± 0.088

a Note that the relaxation rates are reported here as times (inverse of rate) because these are more intuitive; they were, however, fitted as rates. Uncertainties were estimated using residual bootstrapping.

The BSA phantom showed no large difference in signals from 2^+^B, 2^−^B, or 3B excitation, suggesting the absence of both ihMT effects and MT asymmetry; the best fit was achieved when constraining δ to be fixed at zero. For PL161, the best fit was obtained when δ was fixed to 1; however, for HC, a slightly improved fit was found when δ was allowed to vary. Uncertainty analysis also showed that some parameter estimates for HC are less precise than the others; this finding reflects instability of the model fitting, but also it should be noted that the residual bootstrapping method used is less valid for the situation with nonrandom structure in the residuals. Supporting Information Figure S2 plots MTR and ihMTR derived from these data.

### 4.3 Choice of imaging parameters for in vivo experiment and imaging results

**Figure 8:**
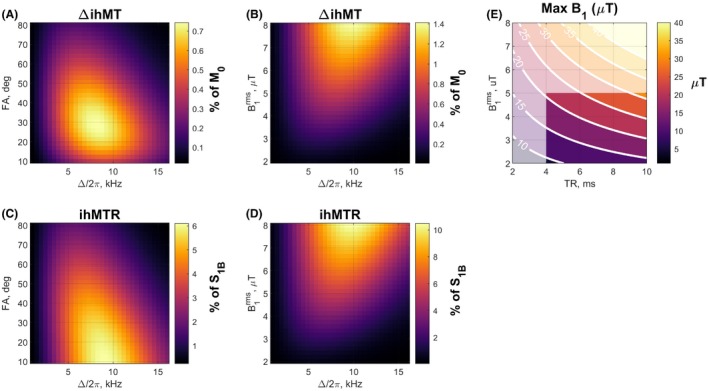
Results of contrast simulations for white matter. A, ΔihMT as a function of Δ and flip angle, for fixed TR = 5 ms, τ = 2 ms, and $B_{1}^{\mathrm{rms}}=5\mu T$. B, ΔihMT as a function of Δ and $B_{1}^{\mathrm{rms}}$ for fixed TR = 5 ms, τ = 2 ms, and flip angle 30°. C,D, ihMTR computed from (A) and (B), respectively. E, Maximum B_1_ (i.e., peak B_1_ in each RF pulse) as a function of TR and $B_{1}^{\mathrm{rms}}$ for fixed τ = 2 ms and flip angle 30°. The shaded regions show inaccessible solutions on our 1.5T system. RF duty cycle limits suggest TR≥2τ and SAR limits mean $B_{1}^{\mathrm{rms}}\leq 5\mu T$. In addition, max B_1_ cannot exceed 20μT

Results from phantom experiments indicated that similar ihMTR can be obtained using both bSSFP and SPGR, but contrast‐to‐noise ratio is higher for the former. We, therefore, focused on bSSFP imaging in vivo and sought to optimize acquisition parameters for the white matter tissue parameters listed in Section 3.2. The steady‐state method has some particular properties: (i) the optimal flip angle to use depends on the steady‐state of the whole system, not just the dipolar component; (ii) hardware limits constrain the achievable parameters but sometimes counterintuitively. Figure 8 illustrates some of these trade‐offs. For example, Figure 8A shows that the peak ΔihMT occurs at flip angle ~30° and offset ~8 kHz for τ = 2 ms and $B_{1}^{\mathrm{rms}}=5\mu T$, which is the maximum allowed within SAR constraints on the MR system used here. Figure 8B shows that increasing the $B_{1}^{\mathrm{rms}}$ would continue to increase the contrast. Parts C and D show ihMTR instead; here the peak value occurs at a lower flip angle but this is because the reference single band image has a lower signal at that flip angle. Setting the parameters to maximize ΔihMT maximizes the effect size relative to the total magnetization; ihMTR in the region of 5% is then expected for white matter.

**Figure 9:**
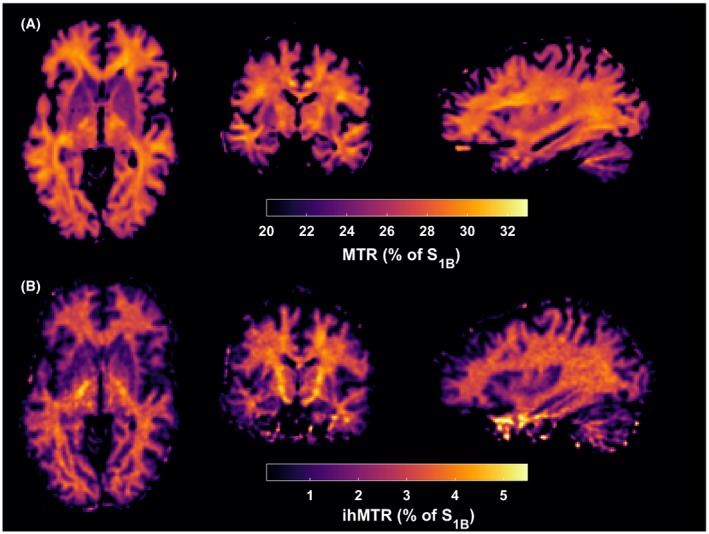
In vivo image results showing MTR (A) and ihMTR (B)

Figure 8E shows that use of single multiband pulses for both saturation and excitation leads to a coupling between hardware constraints; to achieve a certain $B_{1}^{\mathrm{rms}}$ for a fixed τ the peak B_1_ needed (i.e., the peak amplitude of the RF pulse) increases as TR increases. This is because reducing TR leads to an increase in $B_{1}^{\mathrm{rms}}$ for a given RF pulse, as the same energy is delivered at a higher rate. Increasing pulse duration reduces this maximum, but also reduces $B_{1}^{\mathrm{rms}}$ and may necessitate an increase in readout bandwidth, affecting signal‐to‐noise ratio. The human imaging protocol was designed based upon these considerations. Figure 9 shows the acquired human brain MTR and ihMTR images. As predicted the ihMTR in white matter is close to 4‐5%, while MTR is nearer to 30%. The images show visibly different contrasts with ihMTR appearing more specific to white matter, and with the corticospinal tracts clearly visible.

## 5 DISCUSSION

This work has demonstrated inhomogeneous MT contrast in steady‐state gradient echo sequences using multiband pulses. Typical ihMT sequences use a saturation preparation step followed by a readout step. The combined multiband approach instead uses the same RF pulse to both excite free water and provide saturation of semisolid magnetization. This study presents theory for modeling the resulting signals, designing the RF pulses, and experimental validation in phantoms and in vivo. The behavior of bSSFP and SPGR sequences is exemplified by Figure 4. As expected, 3B pulses (on‐resonance and dual off‐resonance) lead to a lower signal than 2B (on‐resonance and single off‐resonance) when a dipolar order term is present despite containing the same overall power. This is because the 2B pulses populate the off‐diagonal entries to $\Omega_{\mathrm{semi}}$ in Equation 4 while 3B pulses close this pathway, leading to more efficient saturation of the Zeeman semisolid magnetization. Human brain imaging results (Figure 9) demonstrate similar contrast to other published ihMTR measurements (e.g., see references 4,6,7,23). The derived signal equations (Equations 5 and 6) predict ihMTR in the region of 4‐5% in white matter using literature tissue parameters for our sequences, and this correlates well with observations.

The mathematical signal model used in this work was developed by Varma and co‐authors over many publications.^1^, ^8^ In addition to explicitly including a sum over frequency components necessary for handling multiband pulses, the contribution of this study is to assemble these into a matrix formalism that also includes transverse components of the free water pool, and to then demonstrate that the same matrix equations used to model steady‐state properties in simpler systems can also be used to model ihMT.

In particular, the commonly used instantaneous pulse approximation was used to simplify the calculations, and accuracy was investigated by comparing with full time integration. It may be expected that this approximation would fail for ihMT because the dipolar relaxation times are typically of the order of a few milliseconds, not much greater than the RF pulse durations or indeed the TRs examined. However, we found that the approximation does hold for SPGR sequences in general, with appreciable differences only when 2B pulses are used with duration $\tau ≳T_{1D}^{s}$. The fact that differences only occur with 2B pulses is a clear indication that this is due to the dipolar terms, because these are excluded for 1B and 3B. It is also reasonable that errors would occur when τ is not small compared with $T_{1D}^{s}$ because we would then not properly account for relaxation of the dipolar pool during saturation. 

For bSSFP, there are much larger discrepancies associated with the instantaneous approximation, however, these largely vanish when the Bieri‐Scheffler correction^18^ is applied to $R_{2}^{f}$ to account for the fact that the magnetization spends less than the full TR period in the transverse plane. Although derived for a standard Bloch equation model, it is perhaps interesting that this correction still holds here. An explanation for this may come from the original study,^18^ which showed that the longitudinal term is only marginally affected. The additional terms in our MT/ihMT model can be thought of as “longitudinal,” because they couple only to the free water longitudinal magnetization and have no transverse components; hence, it is reasonable that they would behave in the same way.

Another contribution of this work is the development of the efficient eigenvector based direct integration method. The method is similar to other approaches proposed for quantitative MT^24^ or CEST,^25^ but to our knowledge is unique in directly computing the steady‐state signal for rapid imaging sequences in this way and could be generalized to other multi‐pool (or single pool) models. Our results indicate that the instantaneous approximation does hold for the relatively short RF pulses used in this work, but it is not guaranteed to for longer saturation pulses. Portnoy and Stanisz^26^ proposed a method to address this issue, which works by integrating Equation 1 through time for specific pulses. A problem is that because this equation is not homogenous, the effect of a long RF pulse cannot be easily written as a compact product of matrices, as there are multiple terms to consider. The “augmented” matrix approach (i.e., Equation 7) provides a way to sidestep this issue to make the effect of any long pulse simply the product of many matrices. Eigenvector decomposition can then be used to obtain the steady‐state directly without forward integration over multiple TR periods. The relative acceleration afforded by direct calculation is proportional to the number of TR periods that would otherwise have to be simulated; this is typically in the hundreds. The high efficiency of the eigenvector based approach could make it a viable simulation route in cases that the instantaneous approximation does not hold.

Phantom experiments showed that this model can achieve good fits to data, with PL161 the most relevant model for ihMT also studied by others^2^, ^3^ an excellent fit to data was achieved with NRMSE = 0.92%. The fitted $T_{1D}^{s}$ = 20.7 ms agrees with that of Swanson et al,^2^ other quantities are not available in literature or are not comparable as our sample was prepared without a T_1_ shortening agent; however, all values appear plausible. Of interest, the estimated $T_{1Z}^{s}$ was in the 100‐ to 200‐ms range for the phantoms with MT effects, which is quite far from the typically assumed 1 s used by many in the literature.

Figure 7 also shows that there is almost no difference between 2^+^B and 2^−^B for PL161, suggesting very little asymmetry of the absorption line. The fits for HC have slightly larger NRMSE (2.4%), primarily due to a poor fit for the SPGR measurements; a potential explanation for this is that this substance may contain extra unmodeled magnetization pools. We generally observed a high degree of degeneracy in fitting for all phantoms with MT effects. In particular, this was true for the T_1_ of free and semisolid compartments, exchange rate, and fraction δ. We hence limit our conclusions only to the fact that the model does fit the observed data well with plausible parameters, and do not claim that this experiment constitutes a proper quantitative measurement of these parameters. An obvious extension to better explore the lineshape properties would be to add measurements at different frequency offsets, for example. Note that, while some parameters were fixed for fitting, the reported solutions were the ones that led to the lowest NRMSE in each case (i.e., as far as could be determined, allowing fixed parameters to vary did not lead to reduced NRMSE).

The use of multiband pulses in this study was motivated by related work that showed that this type of RF excitation can stabilize gradient echo based relaxometry by equalizing MT effects between measurements.^10^ Teixeira et al have shown that estimated T_1_ is a function of $B_{1}^{\mathrm{rms}}$ (see fig. 7 in Teixeira et al^10^). An extension to that work incorporating the theory developed here will be to investigate whether changes in apparent T_1_ when using 2B and 3B pulses can be related to ihMT parameters. Similar “pseudo‐quantitative” work from Geeraert et al^27^ suggests this is a possibility.

In addition to the potential for steady‐state relaxometry, rapid gradient echo sequences, particularly bSSFP, offer high signal‐to‐noise ratio per unit time and can generate high $B_{1}^{\mathrm{rms}}$ using relatively short RF pulses because these are repeated rapidly with short TR. The ihMT effect is small; Figure 8 suggests that the contrast in absolute terms should be approximately 0.7% of the total available magnetization (M_0_), for example. This is limited by $B_{1}^{\mathrm{rms}}$, which increases directly with SAR; indeed, one motivation for conducting experiments at 1.5T is that these scanners can typically access much larger $B_{1}^{\mathrm{rms}}$ than higher field systems.

Measured ihMTRs by our method are of the order of a few percent, which are lower than those measured by other methods,^7^ although this might largely be explained by a factor of 2 difference between the definition of ihMTR used in this study and in other works (see for example, Girard et al^6^). Regardless, the measurements are not directly comparable to those from other methods because ihMTR depends also on the signal value in the 1B image, which depends on the relaxation properties of the free water pool, and to a smaller extent on‐resonance MT effects. A direct comparison of efficiency between steady‐state and other existing approaches will constitute future work. Proposed methods to boost contrast‐to‐noise ratio, such as cycling to higher RF power when the low k‐space frequencies are sampled^7^ or the observation that low duty‐cycle pulsed saturation^23^ can enhance contrast, will also be explored for the multiband gradient echo sequences used in this work.

## 6 CONCLUSIONS

Steady‐state imaging sequences generating inhomogeneous MT contrast have been demonstrated using spatially nonselective multiband RF excitation pulses. A matrix‐based approach for quantifying steady‐state signals was developed and demonstrated to provide good fits to phantom data, and to make good predictions for observed contrast in in vivo human brain images using balanced SSFP.

## Supporting information


**FIGURE S1** Top,ΔihMT for bSSFP and SPGR as a function of flip angle for the δ=1 case plotted in Figure 4. Here ΔihMT is shown as a percentage of the overall M_0_ which was arbitrarily fixed as 1.0 in this work. Middle, ihMTR for the same data, here shown as a percentage of the signal from the equivalent single band image (i.e. % of S_1B_). Close to 10% is achieved for both bSSFP and SPGR at lower flip angles. Bottom, the multiband pulses used in this work were computed to maintain the total $B_{1}^{\mathrm{rms}}$; hence, increasing the on‐resonance flip angle results in a reduction of the total off‐resonance saturation power (i.e., $\left\langle B_{1}^{2}\left(\Delta \neq 0\right)\right\rangle$) that creates ihMT contrast. This plot shows ${\surd \langle B}_{1}^{2}\left(\Delta \neq 0\right)\rangle$ for the flip angles used to make this figure (and Figure 4) showing that the effect is small for this sequence. Note that the pulse properties in this simulation were similar, although not exactly the same as those used in vivo. The reader should be aware that the on‐resonance $B_{1}^{\mathrm{rms}}$ (i.e. ${\surd \langle B}_{1}^{2}\left(\Delta =0\right)\rangle$) is larger than the gap between the curve and the 5μT line because the RMS values add in quadrature
**FIGURE S2** MTR and ihMTR as a function of flip angle, derived from the data plotted in Figure 7; please note that these plots use separate y‐axes for MTR and ihMTR because of their different ranges. For the two ihMT phantoms (PL‐161 and HC), the MTR is much larger than ihMTR. BSA shows large MTR but small ihMTR; a small degree of ihMTR is seen for BSA at high flip angles with the SPGR data but this could not be explained easily by model fitting. Nonzero ihMTR observed in water was attributed to errors from off‐resonance related to an air bubble
**TABLE S1** Upper and lower fit bounds (UB/LB) for each parameter

## APPENDIX 1

Equations 2 and 4 as presented made some simplifying assumptions about equivalent relaxation and exchange rates between semisolid pools, and equivalent absorption lineshapes for these pools. The more general formulation for Λ would be:

(A1) $$\Lambda \;=\;\left(\begin{array}{cccccc} -R_{2}^{f} & \Delta \omega & 0 & 0 & 0 & 0\\ -\Delta \omega & -R_{2}^{f} & 0 & 0 & 0 & 0\\ 0 & 0 & -\left(k_{1}\left(1-\delta \right)+k_{2}\delta \right)M_{0}^{s}-R_{1}^{f} & k_{1}M_{0}^{f} & k_{2}M_{0}^{f} & 0\\ 0 & 0 & k_{1}\left(1-\delta \right)M_{0}^{s} & -k_{1}M_{0}^{f}-R_{1Z}^{s1} & 0 & 0\\ 0 & 0 & k_{2}\delta M_{0}^{s} & 0 & -k_{2}M_{0}^{f}-R_{1Z}^{s2} & 0\\ 0 & 0 & 0 & 0 & 0 & -R_{1D}^{s2} \end{array}\right)$$

where *k_1_* and *k_2_* are the exchange rates for semisolid pools s1 and s2, respectively, and $R_{1Z}^{s1}$ and $R_{1Z}^{s2}$ are the (Zeeman order) longitudinal relaxation rates for these pools. $R_{1D}^{s2}$ is the dipolar order longitudinal relaxation rate, now only for pool s2. The RF interaction matrix Ω consists of free and semisolid pool sub‐matrices and only the latter would be altered to:

(A2) $$\Omega_{\mathrm{semi}}\;=\;\sum_{j\;=\;1}^{N_{\mathrm{bands}}}\left(\begin{array}{ccc} -W_{j}^{1} & 0 & 0\\ 0 & -W_{j}^{2} & W_{j}^{2}\;\frac{\Delta_{j}^{\prime }}{\omega_{\mathrm{loc}}}\\ 0 & W_{j}^{2}\;\frac{\Delta_{j}^{\prime }}{\omega_{\mathrm{loc}}} & -W_{j}^{2}{\left(\frac{\Delta_{j}^{\prime }}{\omega_{\mathrm{loc}}}\right)}^{2} \end{array}\right).$$

Here the absorption rate $W_{j}^{1,2}=\pi \gamma^{2}{\left|b\{\Delta_{j}\}\right|}^{2}g_{1,2}\left(\Delta_{j}^{\prime },\;T_{2}^{s1,2}\right)$, is now made explicitly different for each pool, because the lineshape functions $g_{1,2}\left(\Delta ,\;T_{2}^{s1,2}\right)$ could be different.
