# Supplementary material for: Steady‐state imaging with inhomogeneous magnetization transfer contrast using multiband radiofrequency pulses
Source: Magn Reson Med. 2019 Sep 19;83(3):935–49. doi: 10.1002/mrm.27984 (PMC6881187; doi:10.1002/mrm.27984)
Supplement: Supplementary file 1 — FIGURE S1 Top,ΔihMT for bSSFP and SPGR as a function of flip angle for the δ=1 case plotted in Figure 4. Here ΔihMT is shown as a percentage of the overall M0 which was arbitrarily fixed as 1.0 in this work. Middle, ihMTR for the same data, here shown as a percentage of the signal from the equivalent single band image (i.e. % of S1B). Close to 10% is achieved for both bSSFP and SPGR at lower flip angles. Bottom, the multiband pulses used in this work were computed to maintain the total B1rms; hence, increasing the on‐resonance flip angle results in a reduction of the total off‐resonance saturation power (i.e., ⟨B12(Δ≠0)⟩) that creates ihMT contrast. This plot shows √⟨B12(Δ≠0)⟩ for the flip angles used to make this figure (and Figure 4) showing that the effect is small for this sequence. Note that the pulse properties in this simulation were similar, although not exactly the same as those used in vivo. The reader should be aware that the on‐resonance B1rms (i.e. √⟨B12(Δ=0)⟩) is larger than the gap between the curve and the 5μT line because the RMS values add in quadrature FIGURE S2 MTR and ihMTR as a function of flip angle, derived from the data plotted in Figure 7; please note that these plots use separate y‐axes for MTR and ihMTR because of their different ranges. For the two ihMT phantoms (PL‐161 and HC), the MTR is much larger than ihMTR. BSA shows large MTR but small ihMTR; a small degree of ihMTR is seen for BSA at high flip angles with the SPGR data but this could not be explained easily by model fitting. Nonzero ihMTR observed in water was attributed to errors from off‐resonance related to an air bubble TABLE S1 Upper and lower fit bounds (UB/LB) for each parameter [file MRM-83-935-s001.docx]

**Steady-state imaging with ihMT contrast using multiband RF pulses: Supporting information**

**Supporting Information Figure S1**


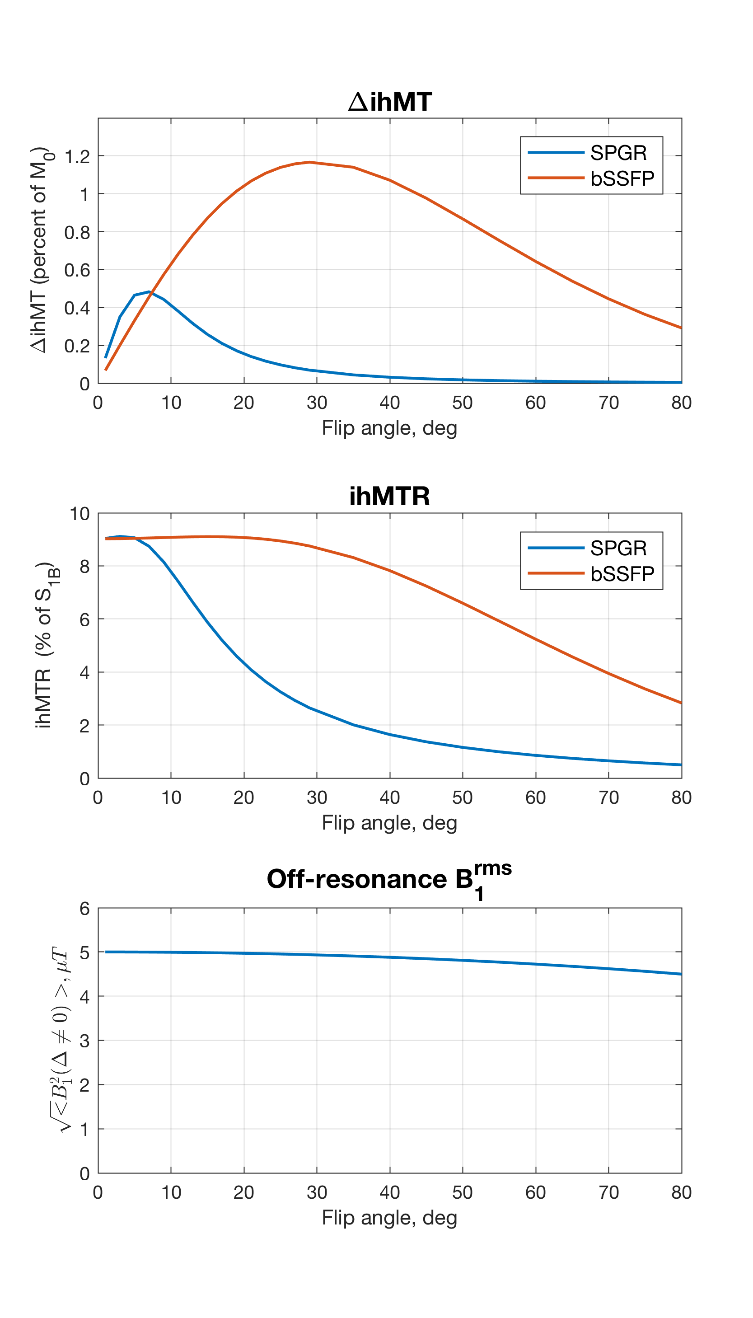


*Top -* $\Delta ihMT$ for bSSFP and SPGR as a function of flip angle for the $\delta=1$ case plotted in Figure 4. Here $\Delta ihMT$ is shown as a percentage of the overall M_0_ which was arbitrarily fixed as 1.0 in this work. *Middle -*  ihMTR for the same data, here shown as a percentage of the signal from the equivalent single band image (i.e. % of S_1B_). Close to 10% is achieved for both bSSFP and SPGR at lower flip angles. *Bottom –* the multiband pulses used in this work were computed to maintain the total $B_{1}^{rms}$; hence increasing the on-resonance flip angle results in a reduction of the total off-resonance saturation power (i.e. ${\langle B}_{1}^{2}(\Delta\neq0)\rangle$) that creates ihMT contrast. This plot shows ${\surd\langle B}_{1}^{2}(\Delta\neq0)\rangle$ for the flip angles used to make this figure (and Figure 4) showing that the effect is small for this sequence. Note that the pulse properties in this simulation were similar though not exactly the same as those used in vivo. The reader should be aware that the on-resonance $B_{1}^{rms}$ (i.e. ${\surd\langle B}_{1}^{2}(\Delta=0)\rangle$) is larger than the gap between the curve and the $5\mu T$ line since the RMS values add in quadrature.


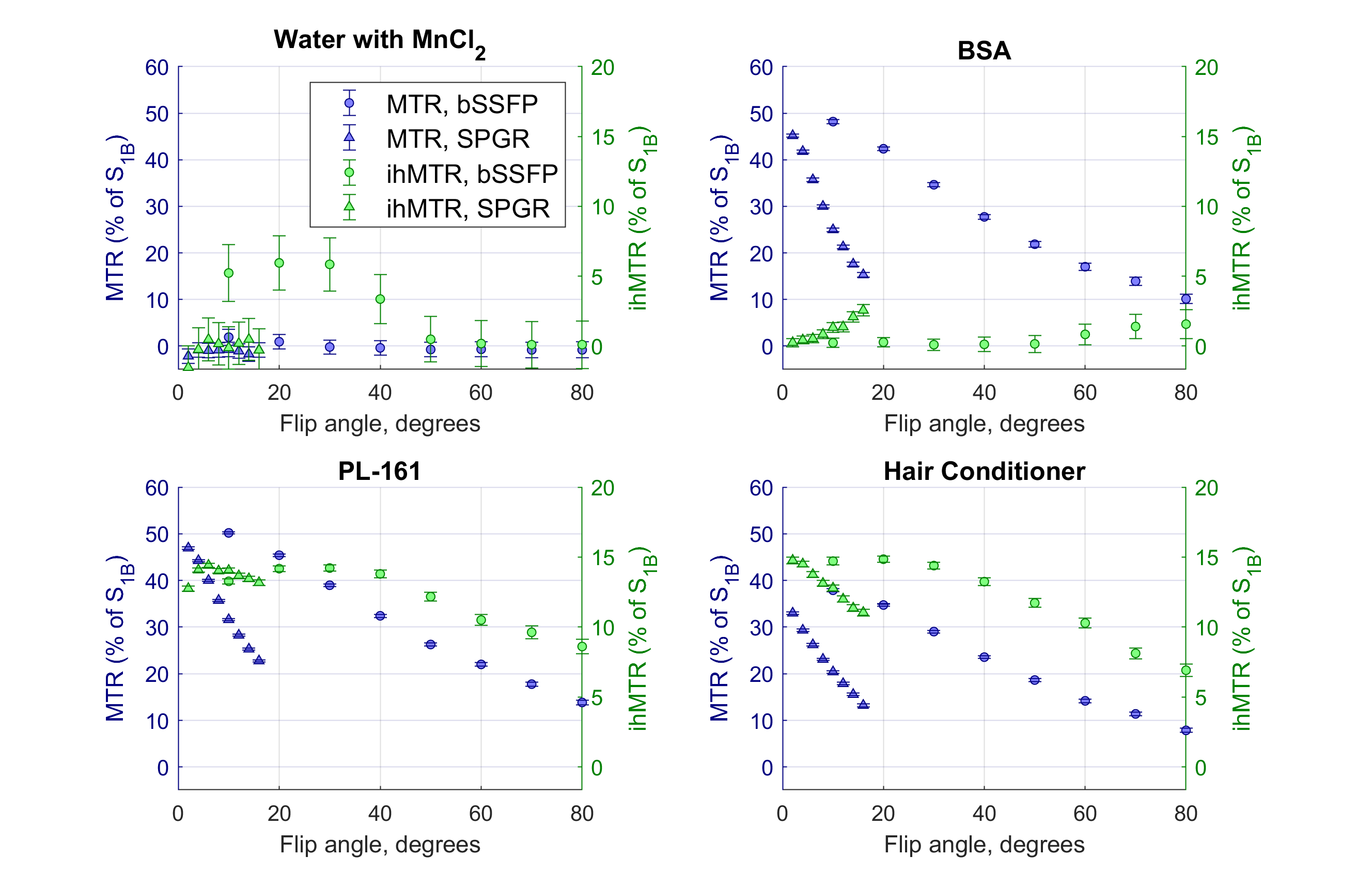


**Supporting Information Figure S2**: MTR and ihMTR as a function of FA, derived from the data plotted in Figure 7; please note that these plots use separate y-axes for MTR and ihMTR because of their different ranges. For the two ihMT phantoms (PL-161 and hair conditioner) the MTR is much larger than ihMTR. Note that non-zero ihMTR observed in water was attributed to errors from off-resonance related to an air bubble. BSA shows large MTR but very little ihMTR; a small degree of ihMTR is seen for BSA at high flip angles with the SPGR data but this could not be explained easily by model fitting.

|  | $\boldsymbol{R}_{\boldsymbol{1}}^{\boldsymbol{f}}$, s^-1^ | | $\boldsymbol{R}_{\boldsymbol{2}}^{\boldsymbol{f}}$, s^-1^ | | $\boldsymbol{M}_{\boldsymbol{0}}^{\boldsymbol{s}}$ | | | | $\boldsymbol{R}_{\boldsymbol{1}\boldsymbol{Z}}^{\boldsymbol{s}}$, s^-1^ | | | $\boldsymbol{T}_{\boldsymbol{2}}^{\boldsymbol{s}}$, $\boldsymbol{\mu s}$ | | | *k*, s^-1^ | | $\boldsymbol{\delta}$ | | $\boldsymbol{R}_{\boldsymbol{1}\boldsymbol{D}}^{\boldsymbol{s}}$, s^-1^ | | |
| --- | --- | --- | --- | --- | --- | --- | --- | --- | --- | --- | --- | --- | --- | --- | --- | --- | --- | --- | --- | --- | --- |
|  | LB | UB | LB | UB | | LB | UB | LB | | UB | LB | | UB | LB | | UB | LB | UB | | LB | UB |
| MnCl_2_ | 0.2 | 3 | 1 | 40 | | 0 | 0 | - | | - | - | | - | - | | - | - | - | | - | - |
| BSA | 0.2 | 3 | 1 | 40 | | 0 | 1 | 0.8 | | 50 | 10 | | 25 | 10 | | 100 | 0 | 0 | | - | - |
| PL161 | 0.2 | 3 | 1 | 40 | | 0 | 1 | 0.8 | | 50 | 10 | | 25 | 10 | | 100 | 1 | 1 | | 30 | 66 |
| HC | 0.2 | 3 | 1 | 40 | | 0 | 1 | 0.8 | | 50 | 10 | | 25 | 10 | | 100 | 0 | 1 | | 30 | 66 |

**Supporting Information Table S1:** Upper and lower fit bounds (UB/LB) for each parameter. Note that the parameters here are quoted as they were actually fitted (i.e. relaxation rates rather than time constants). If a parameter was fixed then the upper and lower bounds are set to be the same; if a parameter is irrelevant because others are fixed, then this is marked with a dash.
